# B-cell–driven relapse and anti-CD20 rescue therapy after Alemtuzumab in RRMS: case report and literature review

**DOI:** 10.3389/fimmu.2025.1710669

**Published:** 2026-01-08

**Authors:** Pietro Antonio Bruno, Stefania Barone, Angelo Pascarella, Pier Luigi Lanza, Emanuele Tinelli, Antonio Gambardella, Paola Valentino

**Affiliations:** 1 Department of Medical and Surgical Sciences, Institute of Neurology, Magna Graecia University, Catanzaro, Italy; 2 Department of Medical and Surgical Sciences, Magna Græcia University of Catanzaro, Catanzaro, Italy; Regional Epilepsy Centre, Great Metropolitan “Bianchi-Melacrino-Morelli” Hospital, Reggio Calabria, Italy; 3 Biocontrol, Diagnostic Imaging Center, Cosenza, Italy; 4 Department of Medical and Surgical Sciences, Institute of Neuroradiology, Magna Graecia University, Catanzaro, Italy

**Keywords:** relapsing–remitting multiple sclerosis, alemtuzumab, ocrelizumab, b cells, steroid-resistant relapse, immune reconstitution therapy, secondary autoimmunity, neuroimmunology

## Abstract

**Background:**

Alemtuzumab is an immune reconstitution therapy (IRT) approved for highly active relapsing–remitting multiple sclerosis (RRMS). Notably, the differential reconstitution of B and T cells after alemtuzumab may trigger paradoxical B–cell–mediated inflammation and secondary autoimmunity.

**Case Report:**

We describe the case of a 44-year-old woman with RRMS who experienced a severe, steroid-resistant relapse nine months after her second alemtuzumab cycle. Lymphocyte subtyping revealed disproportionate B-cell repopulation, suggesting a B-cell–mediated immunopathogenesis. Early ocrelizumab switch resulted in rapid clinical and radiological recovery and sustained stability.

**Literature review:**

We conducted a narrative review of early and paradoxical disease reactivation after alemtuzumab, identifying common features: early onset, poor steroid response, large or tumefactive lesions, and marked B-cell hyperrepopulation. Anti-CD20 therapy often induced rapid remission. These findings may suggest a distinct B-cell–driven mechanism of inflammation. Prior exposure to fingolimod has been observed in several cases, but does not uniformly account for the observed phenotype.

**Conclusion:**

This case contributes to the growing evidence that a subset of patients may experience B-cell–driven inflammatory reactivation after alemtuzumab treatment. Lymphocyte subtyping should be performed, and a predominance of CD19^+^ B cells can guide a timely therapeutic switch to anti-CD20 therapy. Further studies are needed to define whether this represents a distinct post-IRT immunopathological entity.

## Introduction

Multiple sclerosis (MS) is a chronic autoimmune disorder characterized by central nervous system (CNS) demyelination and neurodegeneration. Genetic and environmental factors jointly contribute to immune tolerance loss in MS (1). Historically, MS pathogenesis has been attributed mainly to CD4^+^ T-helper 1 (Th1)-driven inflammation, with Th2 cells serving a counter-regulatory role. However, increasing evidence highlights the critical involvement of B lymphocytes in MS through antigen presentation, autoantibody production, and the formation of ectopic follicle-like structures in the meninges. Additionally, indirect evidence of their contribution comes from the effectiveness of B-cell-based immunotherapies (2). This evolving understanding of MS immunopathology has led to the development of disease-modifying therapies (DMTs) targeting specific immune subsets. Among these, immune reconstitution therapies (IRTs) such as alemtuzumab aim for long-term disease control through profound yet temporary lymphocyte depletion followed by immune repopulation. Alemtuzumab, a humanized anti-CD52 monoclonal antibody, targets both T and B cells. While generally effective, outcomes may vary depending on the pattern of immune reconstitution, especially the balance between T and B cell recovery (3). Post-alemtuzumab autoimmunity affects up to 40–50%. Its pathophysiology remains complex and multifactorial but seems to involve a breakdown in regulatory networks during immune reconstitution, favoring autoreactive B-cell clones (4).

### Case presentation

**Figure 1:**
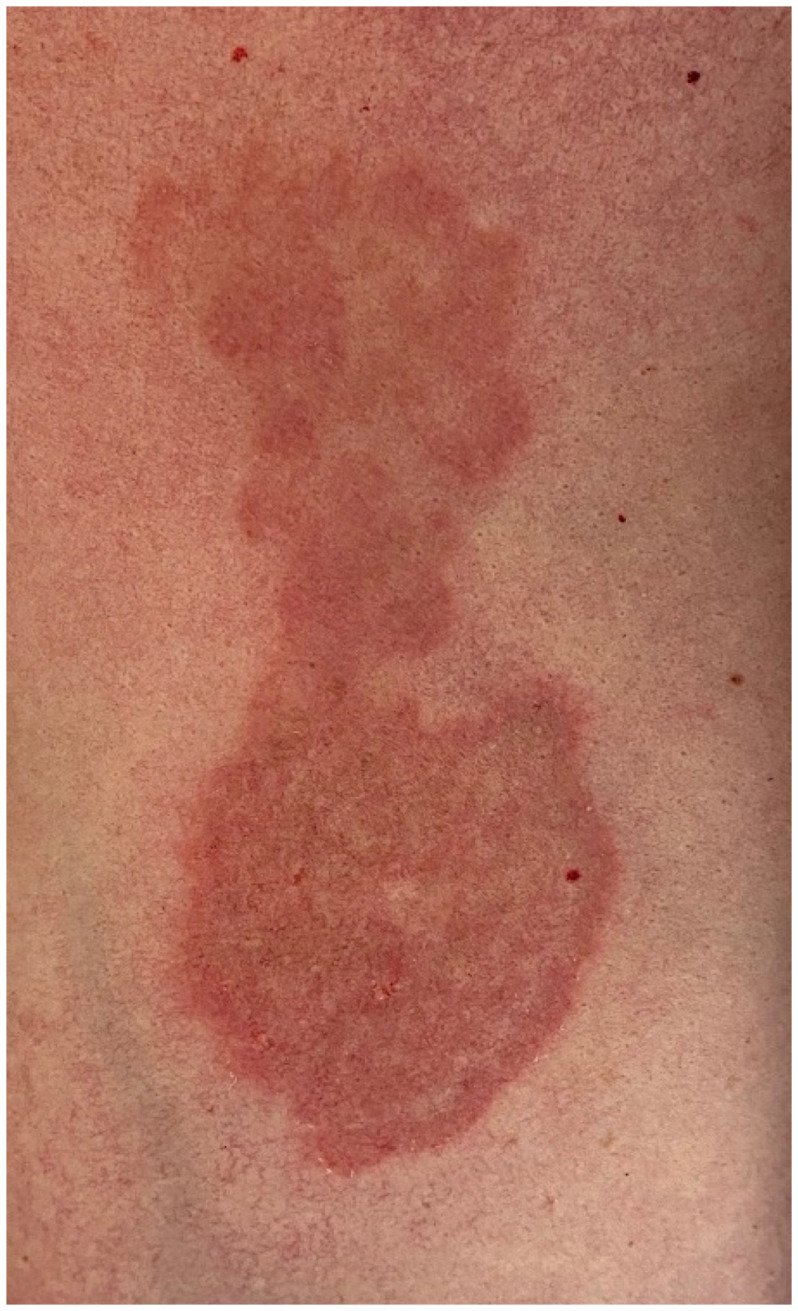
Erythematous lesion in the left breast region, diagnosed as Tinea Corporis and tested positive on culture for Microsporum Canis.

**Figure 2:**
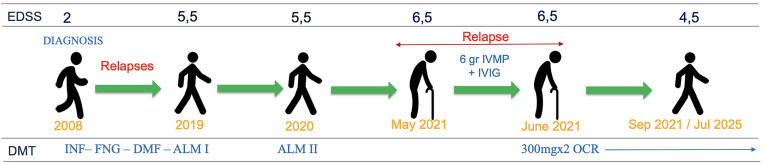
Clinical timeline illustrating treatments, relapses, and EDSS progression. EDSS, Expanded Disability Status Scale; IVMP, intravenous methylprednisolone; IVIG, intravenous immunoglobulins.

**Figure 3:**
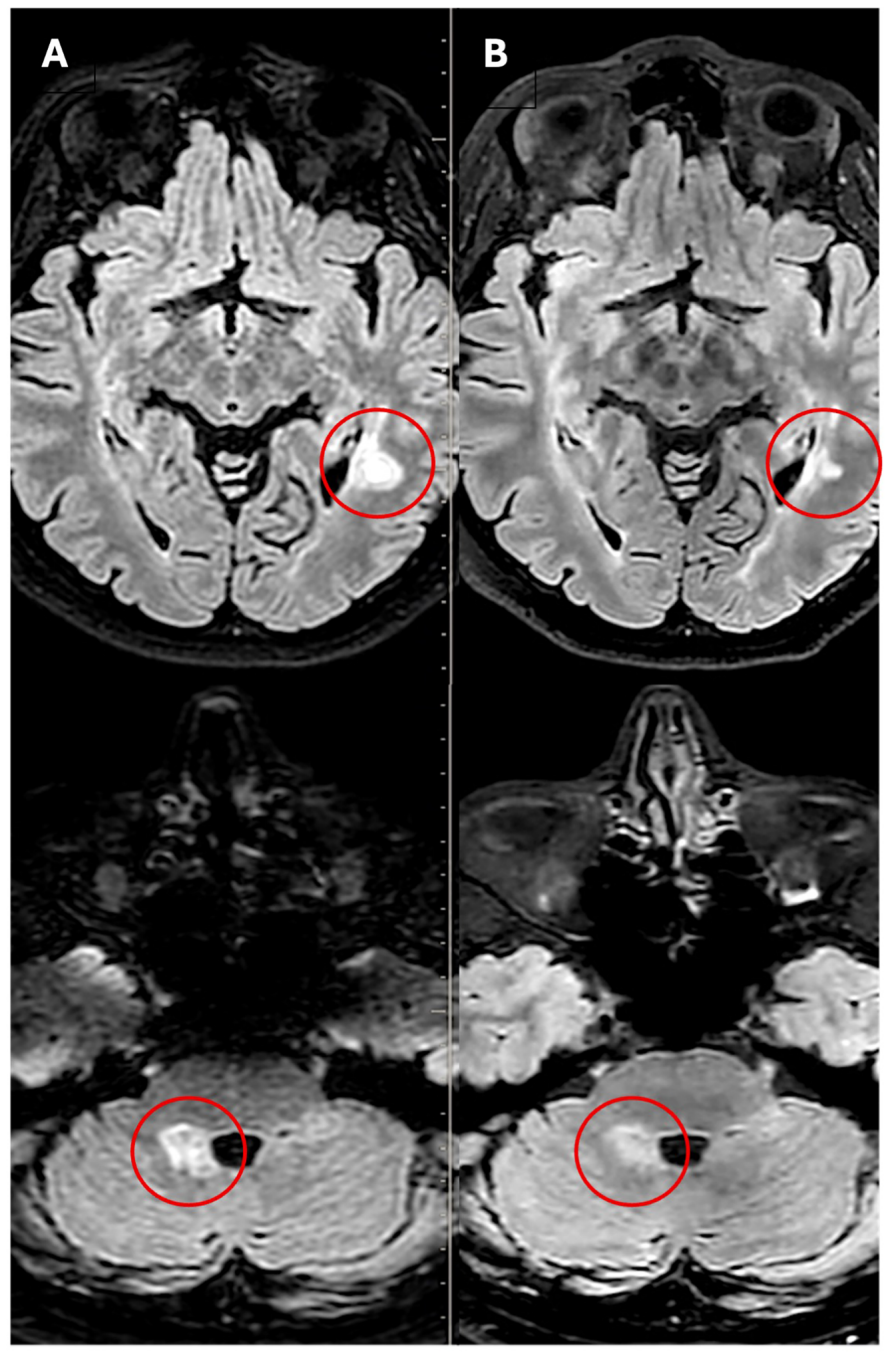
On the left **(A)**, axial FLAIR brain MRI reveals a large demyelinating lesion extending from the globose nucleus to the middle and superior cerebellar peduncle, with partial peripheral contrast enhancement on post-gadolinium T1-weighted images (not shown). An additional lesion is seen in the left posterior temporal white matter, also showing contrast enhancement on post-gadolinium T1-weighted images (not shown). On the right **(B)**, follow-up axial FLAIR brain MRI after Ocrelizumab therapy shows a reduction in size of both previously enhancing lesions, with resolution of contrast enhancement on post-gadolinium T1-weighted images (not shown). MRI was performed using 1.5-T and 3.0-T scanners with sequences including TSE-T2, FLAIR 3D, FFE-T2, 3D-T1, DWI, and STIR.

A 44-year-old woman with asthma and a history of thyroidectomy for multinodular goiter was diagnosed with RRMS in 2008 after presenting with weakness and hypoesthesia in the lower limbs. The diagnosis was established according to the 2005 McDonald diagnostic criteria (5), based on clinical and MRI findings, supported by the presence of cerebrospinal fluid (CSF) oligoclonal bands and negative cytology. Although formulated earlier, the diagnosis fully aligns with current 2024 McDonald revision criteria (6). Differential diagnoses were excluded through an extensive infectious and autoimmune work-up, all negative. Over the following years, she experienced multiple relapses involving visual and sensory symptoms, treated with intravenous methylprednisolone (IVMP) with only partial recovery, leading to progressive disability accumulation. She received sequential disease-modifying therapies (DMTs), including interferon beta-1a, fingolimod, and dimethyl fumarate, all discontinued due to persistent clinical and radiological activity. Despite treatment, her Expanded Disability Status Scale (EDSS) worsened to 5.5 by April 2019. Alemtuzumab was initiated that month, followed by a second cycle in July 2020. In April 2021—nine months after the second cycle—she developed a severe steroid-refractory relapse accompanied by a cutaneous fungal infection (Tinea corporis, Microsporum canis positive; **Figure 1**). Neurological examination showed gait ataxia, dysarthria, mild dysphagia, and incontinence, with EDSS worsening to 6.5. The overall disease course, treatment timeline, and key clinical milestones are summarized in **Figure 2**. Brain MRI revealed a large demyelinating lesion extending from the globose nucleus to the middle and superior cerebellar peduncle and fourth ventricle, with incomplete peripheral enhancement (**Figure 3A**). After 11 days of IVMP (500 mg/day), no meaningful improvement was observed; EDSS remained 6.5, although the patient reported slight improvements in speech and gait after starting rehabilitation. Lymphocyte subset analysis by multiparametric flow cytometry (Navios, Beckman Coulter) using anti-CD45 monoclonal antibodies demonstrated marked B-cell expansion (CD19^+^ 39.6%, 515 cells/µL; normal range 7–23%), with relative CD4^+^ and CD8^+^ T cell depletion. Results were expressed as both percentages and absolute counts (cells/µL) (**Table 1**). The sample was collected immediately before corticosteroid administration and analyzed at the Flow Cytometry Service, Magna Graecia University of Catanzaro. These findings suggested a B-cell–driven relapse. A short course of intravenous immunoglobulins (IVIG) was attempted but discontinued on day 3 because of side effects and lack of benefit. Given the immunophenotype and clinical status, an early switch to ocrelizumab was made. Ten days before treatment initiation, follow-up MRI still showed active enhancement, with a new lesion in the left posterior temporal white matter. The first 300 mg ocrelizumab infusion was administered in June 2021, followed by a second dose 15 days later. Repeat lymphocyte profiling before the second dose showed complete CD19^+^ B cell depletion (0.0%, 0 cells/µL) and normalized CD4^+^/CD8^+^ T-cell ratios (**Table 1**). The patient demonstrated rapid clinical improvement. MRI in September 2021 confirmed resolution of enhancement and reduction in lesion burden (**Figure 3B**). EDSS improved to 4.5 with near-complete symptom recovery. She continued ocrelizumab 600 mg every six months, with the latest infusion in July 2025. No relapses or MRI activity were recorded, confirming long-term disease stability (51-month follow-up). Longitudinal monitoring showed sustained CD19^+^ B-cell suppression. A moderate reduction in serum immunoglobulin was observed [IgG: 6.02 g/L (n.v. 7.00–16.00), IgM: <0.24 g/L (n.v. 0.40–2.50)], along with mild hypogammaglobulinemia at serum protein electrophoresis [gamma: 8.8% (n.v. 11.1–18.8%)]. During the third year of treatment, she developed a single herpes zoster episode, which resolved completely with antivirals.

**Table 1:** Lymphocyte subset distribution before and after Ocrelizumab treatment.

CD	Lymphocytes types	Pre-Ocrelizumab	Post-Ocrelizumab	Normal range
CD45+	Lymph. absolute counts	1301 cell/µL	982 cell/µL	–
CD3+	Lymph. T Tot.	32.3% 420 cell/µL	82.5% 810 cell/µL	61–85%
CD3+CD4+	Lymph. T Helper/Inducer	13.1% 170 cell/µL	38.2% 375 cell/µL	28–58%
CD3+CD8+	Lymph. T Cytotoxic/Suppressor	17.1% 223 cell/µL	29.3% 287 cell/µL	19–48%
CD3-CD16+CD56+	Cell. NK	26.6% 346 cell/µL	16.9% 166 cell/µL	9–21%
CD3+CD16+CD56+	Cell. NKT	2.2% 29 cell/µL	3.5% 34 cell/µL	–
CD19+	Lymph. B Tot.	**39.6% 515 cell/µL**	**0.0% 0 cell/µL**	7–23%

Bold values indicate the lymphocyte subsets of main interest, highlighting CD19+ B-cell over-repopulation during relapse and their rapid depletion after anti-CD20 therapy.

This case report was prepared in accordance with the CARE (CAse REport) Guidelines (7), ensuring comprehensive and transparent reporting of clinical data, investigations, and outcomes.

## Literature review

Due to the increasing reports of paradoxical disease reactivation after Alemtuzumab, we performed a narrative literature review to put our observations into context. Searches were conducted in PubMed and Scopus using the terms “Alemtuzumab, “ “multiple sclerosis, “ “relapse, “ and “paradoxical reactivation. “ Only English-language case reports and series that described new clinical or radiological activity following Alemtuzumab treatment in RRMS were included. This review aimed to identify common clinical and immunological patterns, such as the timing of reactivation, MRI features, treatment responses, and lymphocyte changes, with a special focus on B-cell repopulation. Nine publications involving 20 patients published since 2017 met these criteria and are summarized in **Supplementary Table S1** (8–16). The key clinical and immunological findings are summarized briefly below. Across the cases, the time from Alemtuzumab administration to disease reactivation ranged from 4 to 24 months, with most relapses occurring within the first year. Only one late case was reported at 24 months. All patients showed new T2 and/or gadolinium-enhancing lesions —often multiple or confluent—accompanying severe clinical worsening, with EDSS scores reaching up to 9.5 during the acute phase (11). Immunologically, most patients exhibited early and disproportionate CD19+ B cell reconstitution, often alongside a reduction in regulatory subsets (CD19+CD24hiCD38hi) or an increase in Th1/Th17 profiles. In one case (Hyun et al., 2019), a progressive decline in regulatory B cells preceded relapse, suggesting a potential predictive biomarker (11). The most common rescue strategy was switching to anti-CD20 therapy (rituximab or ocrelizumab), often after administering intravenous steroids or plasma exchange. In nearly all cases, this approach resulted in rapid clinical and radiological stabilization. A recurring pattern in over half of the cases was prior use of fingolimod before starting alemtuzumab. This raised suspicions of a lymphocyte sequestration effect that might interfere with alemtuzumab’s mechanism of action. Willis et al. (2017) systematically explored this phenomenon in an observational study, in which nine patients experienced early and significant disease activity within 12 months after switching from fingolimod to alemtuzumab. The authors proposed that autoreactive lymphocytes sequestered in secondary lymphoid organs during fingolimod treatment may have evaded alemtuzumab-mediated depletion, re-entered circulation after the drug’s biological window closed, and triggered disease reactivation (15).

## Discussion

This case highlights an unusually severe disease reactivation occurring shortly after the second Alemtuzumab cycle. The relapse showed a steroid-refractory course and a disproportionate CD19^+^ B-cell expansion. Although Alemtuzumab typically induces long-term remission through transient lymphocyte depletion followed by immune reconstitution, growing evidence suggests that, in some individuals, this process may lead to dysfunctional immune reconfiguration. According to Rolla et al., Alemtuzumab induces prolonged lymphopenia lasting several years despite its short plasma half-life. Its therapeutic efficacy depends not only on lymphocyte depletion but also on how the immune system reconstitutes itself. While Alemtuzumab rapidly depletes CD4^+^, CD8^+^, and B cells, recovery times vary among these subsets. Mature naive CD19^+^ B cells initially decline by over 85%, then an overexpansion of immature B cells occurs—up to 160–180% of baseline—within 3–6 months post-treatment (17). Recent work by von Essen et al. (2023) describes an immune landscape after alemtuzumab characterized by exhausted T cells, increased regulatory control over proinflammatory T cells, and diminished B-cell regulation driven by elevated Tfh/Tfr ratios (4). These findings align with the broader phenomenon of secondary autoimmunity after Alemtuzumab, which affects up to 48% of treated patients and is largely B-cell driven, as seen in autoimmune thyroiditis, immune thrombocytopenic purpura, and encephalitis (18). The timing and frequency of these adverse events support the idea that B-cell reconstitution can occur without adequate regulatory balance, allowing autoreactive clones to expand. In our patient, the expansion of CD19+ B cells without T-cell proliferation likely reflected such dysregulation and contributed to inflammation. This hypothesis is further supported by the lack of response to acute glucocorticoid treatment during relapse. According to Berkovich (2016) (3), the main action of glucocorticoids is to induce apoptosis in T lymphocytes, reducing their infiltration into the CNS. The patient’s rapid and sustained recovery after anti-CD20 therapy, which specifically depletes B cells, supports this view. Meltzer et al. (2020) proposed a proactive approach targeting B cells when CD19^+^ counts reach 40–50% of baseline, using low-dose rituximab (“whack-a-mole” approach), successfully preventing autoimmune complications (19). Although anti-CD20 therapy in our case was not preventive, the notable expansion of CD19+ B cells after the second alemtuzumab cycle and the positive response to ocrelizumab highlight the pathophysiological relevance of this model and support a personalized, immunophenotype-driven treatment strategy. Our review of 20 patients across nine reported cases confirmed that early post-alemtuzumab relapses typically show a pattern of rapid B-cell repopulation, steroid resistance, severe clinical and radiological worsening (EDSS up to 9.5 during the acute phase (11)), and response to anti-CD20 therapy. This raises the possibility that these relapses represent a distinct, B-cell–mediated inflammatory pattern emerging within the post-alemtuzumab immune landscape. Notably, Rinaldi et al. (2018) provided direct evidence of B-cell dysregulation in the CNS after alemtuzumab, including a shift in oligoclonal band mirror patterns suggestive of peripheral and intrathecal B-cell clonal expansion (16).

An additional consideration is the potential impact of prior fingolimod therapy. Although previous fingolimod treatment is often observed in patients with post-alemtuzumab disease reactivation, its pathogenic role remains unclear. Some observational reports suggest a ‘sequestration’ mechanism in early-switch scenarios after post-switch reactivation (15). However, data from a large Italian real-world cohort showed a reduction in relapse rate and MRI activity after switching from fingolimod to alemtuzumab, with no evidence indicating a detrimental effect related to the switch itself (20). This finding argues against a consistent pathogenic role for prior fingolimod treatment in subsequent alemtuzumab failure.

To our knowledge, this report represents the longest documented follow-up of a post-alemtuzumab B-cell–dominated relapse successfully treated with Ocrelizumab. The case features detailed immunophenotypic characterization over time and demonstrates sustained CD19^+^ B-cell depletion, long-term safety, and clinical stability over 51 months.

## Limitations

Some methodological limitations should be acknowledged. Extended immunophenotyping, including B-cell subset and cytokine profiling, was not performed; however, both absolute and relative lymphocyte data consistently support a B-cell–driven reactivation pattern. Testing for anti-AQP4 and anti-MOG antibodies was not conducted, as neither the clinical course nor MRI findings indicated features typical of NMOSD or MOGAD, and the overall presentation remained consistent with an RRMS relapse. Lymphocyte subtyping before Alemtuzumab initiation was unavailable, preventing longitudinal comparison across all treatment phases; nonetheless, the immunophenotyping obtained during relapse and after anti-CD20 therapy offers meaningful temporal evidence of causality. Despite these limitations, the integration of immunological, radiological, and long-term clinical data enhances the robustness of the findings reported.

## Conclusion

Lymphocyte subtyping can provide essential insights into treatment failure after IRT. In line with Valis et al. (10), we suggest conducting B- and T-cell subtyping in cases of Alemtuzumab treatment failure and considering an early switch to anti-CD20 therapy when a significant B-cell overrepresentation is observed.

The set of features seen in this case—early B-cell expansion, severe clinical deterioration, corticosteroid resistance, prominent MRI enhancement, and a positive response to anti-CD20 therapy—may indicate a B-cell–dominant reactivation pattern that differs from traditional MS relapse. Further research combining clinical, immunological, and biomarker data is needed to better understand this phenomenon and to improve patient-specific monitoring and management strategies.

This case, consistent with existing research, supports the use of immunophenotyping in patients exhibiting early or severe post-alemtuzumab activity. Personalized immunomonitoring should become a standard part of managing complex MS cases with unusual post-IRT relapses.

## Data Availability

The raw data supporting the conclusions of this article will be made available by the authors, without undue reservation.
