# Supplementary material for: B-cell–driven relapse and anti-CD20 rescue therapy after Alemtuzumab in RRMS: case report and literature review
Source: Front Immunol. 2026 Jan 8;16:1710669. doi: 10.3389/fimmu.2025.1710669 (PMC12825221; doi:10.3389/fimmu.2025.1710669)
Supplement: Supplementary file 1 [file Table1.docx]

**Supplementary Table S1. Clinical, radiological, immunological, and therapeutic features of reported post-Alemtuzumab relapses in RRMS**

| Author (year) | Sex / Age | Time from ALM to relapse (months) | Immunophenotyping findings | MRI characteristics | Rescue therapy | Outcome / Follow-up |
| --- | --- | --- | --- | --- | --- | --- |
| **Haghikia et al., 2017** | M 41 / F 25 | 5–11 m (1st cycle) | Not available | ≥ 20 Gd+ ring-enhancing lesions | IVMP + PLEX + RTX | Marked improvement, FU 9 m |
| **Willis et al., 2017** | 9 pts (5 F / 4 M, median 22 y) | 4.5 m median (1st cycle) | Not available | Multiple T2 or Gd+ lesions (up to 13) | ALM 2nd cycle | Variable response, FU 20 m median |
| **Wehrum et al., 2018** | 3 pts (2 F / 1M, 21–37 y) | 6 m (1st cycle) | CD19↑ in PB | Numerous ring-enhancing lesions (up to 60) | IVMP ± PLEX + RTX | Stable, FU 12–18 m |
| **Rinaldi et al., 2018** | F 27 | 4 m (1st cycle) | CD20+ reactive CSF | Brain + spinal Gd+ lesions | PLEX + IVMP + aHSCT | Clinical improvement |
| **Hyun et al., 2019** | M 20 | 10 m (1st and 2nd cycles) | CD19↑, Breg CD24hiCD38hi↓ in PB | New Gd+ brain lesions | IVMP + PLEX | Partial recovery |
| **Brannigan et al., 2020** | M 39 | 9 m (1st cycle) | Not available | DWI+ lesions, no Gd+ | IVMP + PLEX + RTX + CFX | EDSS 9.5 → 3.5, FU 24 m |
| **Vališ et al., 2020** | F 37 | 4 m (1st cycle) | CD19↑ × 3.4, mem B↑ in PB | Gd+ brain lesions | IVMP + OCRE | Stable ≥ 6 m |
| **Adamec et al., 2022** | F 26 | 9 m (2nd cycles) | CD20+↑ in PB | > 20 brain + spinal Gd+ lesions | IVMP + PLEX + OCRE | Improved, FU 24 m |
| **Rabaneda-Lombarte et al., 2024** | F 30 | 24 m (2nd cycles) | CD19↑, Th1/Th17↑ in PB | TDL + new Gd+ lesions | IVMP + NTZ | Stable, FU ≥ 3 m |
| **Bruno et al. (present case)** | F 44 | 9 m (2nd cycles) | CD19↑ (39.6%, 515 cells/µL) | New Gd+ brain lesions | IVMP + IVIg + OCRE | Marked improvement, FU 51 m |

**Legend:** ALM = Alemtuzumab; IVMP = Intravenous methylprednisolone; PLEX = Plasma exchange; RTX = Rituximab; OCRE = Ocrelizumab; NTZ = Natalizumab; CFX = Cyclophosphamide; aHSCT = Autologous hematopoietic stem cell transplantation; PB = Peripheral blood; Breg = Regulatory B cells; Gd+ = Gadolinium-enhancing lesion; TDL = Tumefactive demyelinating lesions; FU = Follow-up; m = months; y = years. Where available, EDSS scores before and after relapse, disease-modifying therapy (DMT) sequence before Alemtuzumab, and washout duration were also reviewed but are not displayed for brevity.
